# When a Touch of Gold Was Used to Heal the King’s Evil

**DOI:** 10.3201/eid2803.AC2803

**Published:** 2022-03

**Authors:** Jean Krugman, Terence Chorba

**Affiliations:** Atlanta, Georgia, USA (J. Krugman); Centers for Disease Control and Prevention, Atlanta (T. Chorba)

**Keywords:** art science connection, emerging infectious diseases, art and medicine, about the cover, Mycobacterium bovis, bacteria, tuberculosis, tuberculosis and other mycobacteria, lymphadenitis, lymph node, therapeutic touch, when a touch of gold was used to heal the king’s evil, angel (coin) of the second reign

**Figure:**
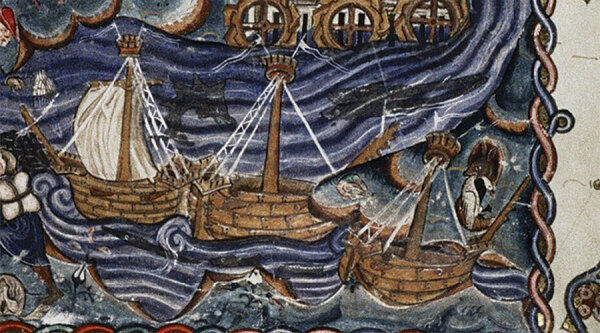
**Angel (Coin) of the Second Reign (1471–1483) of Edward IV.** Gold, 29 mm, 5.1 g, 1473‒1477. British Museum, London, UK.

“'Tis called the evil.

A most miraculous work in this good king,

Which often since my here-remain in England

I have seen him do. How he solicits heaven,

Himself best knows, but strangely visited people,

All swoll’n and ulcerous, pitiful to the eye,

The mere despair of surgery, he cures,

Hanging a golden stamp about their necks,

Put on with holy prayers.”

‒-Malcolm, Act 4, Scene 3, in William Shakespeare’s Macbeth

Throughout history, divine approval has been claimed by many rulers in establishing legitimacy of their monarchy and has been integral to governance in the development of many cultures. In ancient and Imperial China, a tradition of a mandate of heaven, as the will of the universe or natural law, was used to justify the position of the ruler. In the Inca Empire, the traditional ruler was considered the progeny of the sun god and in that capacity had to be accorded absolute power over the people, such as the sun itself has. European history is replete with similar traditions of monarchical claims for legitimacy. In Britain and in France, the evolution of the concept of “the divine right of kings” and the resultant philosophic traditions favoring or opposing such a concept shaped much of the history of the past millennium. Both monarchies claimed to rule by divine will, and to this day, the British Coronation service includes a sacred anointing of the new king or queen.

Many religious traditions have had thaumaturgic (relating to supernatural powers) touch as a tradition. In Britain, reference to the monarch as having divine power in “the royal touch” dates to the 11th century, when it was believed that Edward the Confessor, last of the Anglo-Saxon kings, possessed powers to heal the sick through some form of laying on of hands. In official ceremonies in his and subsequent reigns, subjects could approach the monarch to seek the imperial touch, hoping to cure their ailments or diseases. For centuries, the disease that most readily lent itself to the occasional appearance of success in this regard was scrofula (i.e., lymphadenitis—most commonly tuberculous cervical lymphadenitis), which would manifest itself with painful and visible sores that could go into remission and even go into resolution, giving the impression of a royally induced cure. Scrofula is a term that has fallen into disuse like many other medical terms in English (e.g., catarrh, ague, quinsy, dropsy, and grippe), principally because of diagnostic advances and more precise disease characterization. However, because of the association of its spontaneous remission with the royal touch, tuberculous lymphadenitis was also called “the king's evil,” and throughout most of the past millennium, its presence in European populations was very common.

In well-orchestrated ceremonies in which a monarch would come into contact with crowds of infected people, the laying on of hands benefited both ruler and subject; it underlined the divinely legitimized authority and power of the monarch and demonstrated accessibility to poor mortals. Common petitioners received hope for a cure of their disease by virtue of the imperial touch. Frequently, from the 15th through the 17th centuries, such supplicants were also given a hammered gold coin, known as an Angel, as a gift. This denomination of coin was introduced to England by King Edward IV in 1465, patterned after a similar coin minted in France, called an *ange* or *angelot.*

An example of a gold Angel is featured on this month’s cover. On the obverse (front) of the coin, there is a winged standing figure of the Archangel Michael slaying a dragon (evil) with a spear, a figure with a mythology dating from the 4th century bce. On the reverse, a central shield is depicted within the ship of state with a thick mast and thick main spar, flanked by the King’s initial (E) and a rose; the stays of the mast emanate from the ship’s masthead below the crow’s nest. A depiction of three ships with similar masts and rigging, found in a Flemish 15th century manuscript, is presented in the Figure. Unlike most other coinage that was hammered by hand as the production method that dominated until 1662, the basic design of the Angel remained constant over two centuries. Commonly, these coins were pierced with a hole at the top through which a cloth or chain could be passed for the coin to be worn around the neck. Touching the coin, which the king himself had touched, was thought to be healing as well. Tales of success and demand for physical contact with the king by persons with scrofula became so great that distribution and receipt of such coins gradually became a substitute for the regal touch itself.

**Figure:**
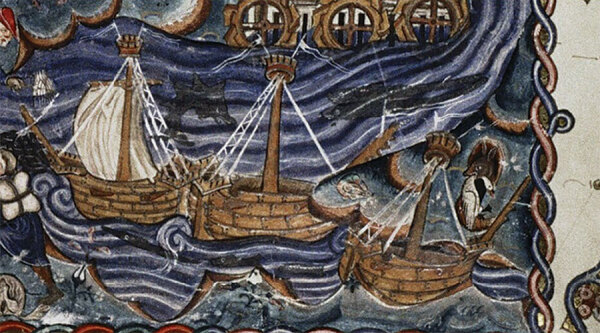
Artist unknown. Magnification of portion of page from Flemish manuscript “Romance of Alexander,” fol. 001r. 15th century. https://digital.bodleian.ox.ac.uk/objects/8d17bc13-14b6-4a56-b3b5-d2e1a935c60d/surfaces/6fb21473-8721-4b74-8916-8519575b90cc/

In March 1882, Robert Koch demonstrated that the tubercle bacillus was the causative organism of the clinical manifestations associated with pulmonary tuberculosis (TB). In 1896, the organism was assigned to a new genus, *Mycobacterium.* At the time, TB was a leading cause of death in Europe; discovery of an infectious etiology led to preventive approaches that resulted in dramatic decreases in cases of illness and death from TB, even in the 19th century. Although lymphadenitis is one of the clinical manifestations of infection with *Mycobacterium tuberculosis*, either as the result of hematogenous or lymphatic spread, cervical lymphadenitis is also often observed when the primary focus of disease is the tonsils or pharynx, as was common in the time of Edward IV, as the result of drinking milk or eating milk products contaminated with bovine tubercle bacilli (i.e., *M. bovis*). Although pasteurization of milk and screening and care of cattle have greatly limited exposure to *M. bovis* since the late 19th century, cervical lymphadenitis remains an extrapulmonary feature of *M. tuberculosis* disease; in 2020, ≈8% of US TB patients had lymphatic TB. Cervical lymphadenitis may also result from receipt of *M. bovis*–derived Bacillus Calmette-Guérin (BCG) vaccine, which is given to infants in high-risk countries to reduce the risk for TB disease and disseminated TB, as well as from nontuberculous (environmental) mycobacterial infections, usually *M. avium intracellulare* or *M. scrofulaceum*. Fortunately for all, the contributions of Koch, his contemporaries, and his successors to our understanding of the infectious etiologies of scrofula have obviated the need to pursue the royal touch of gold to cure the king’s evil.
